# New biotechnological tools to accelerate scab-resistance trait transfer to apple

**DOI:** 10.1590/1678-4685-GMB-2016-0043

**Published:** 2017-02-13

**Authors:** Roberta Cusin, Luís Fernando Revers, Felipe dos Santos Maraschin

**Affiliations:** 1 Plant Physiology Laboratory, Departamento de Botânica, Instituto de Biociências, Universidade Federal do Rio Grande do Sul, Porto Alegre, RS, Brazil.; 2 Laboratory of Plant Molecular Genetics, Centro Nacional de Pesquisa de Uva e Vinho, Empresa Brasileira de Pesquisa Agropecuária, Bento Gonçalves, RS, Brazil.

**Keywords:** Malus, Venturia inaequalis, biotic stress, transformation

## Abstract

Apple is a fruit crop cultivated worldwide. Apple orchards are exposed to a diverse set of environmental and biological factors that affect the productivity and sustainability of the culture. Many of the efforts and costs for apple production rely on reducing the incidence of fungal diseases, and one of the main diseases is apple scab caused by the fungus *Venturia inaequalis*. The economic impact of scab on apple productivity has guided many breeding programs to search for cultivars resistant to apple scab. Introgression from wild relatives has been successful to some extent, and genetic engineering for resistant cultivars has even been employed. This review presents the techniques used to the present time to obtain pathogen-resistant apple cultivars and introduces new biotechnological approaches based on plant plasmids that show promising results for delivering genetic traits with a short-term perspective.

## Introduction

The cultivated apple belongs to the Rosaceae family, Maloideae subfamily, and the farmed apple tree is known as *Malus x domestica* Borkhausen (Harris *et al.*, 2002). Apple orchards are susceptible to several pathogens. The main diseases that affect apple production include apple scab, fire blight, powdery mildew, and the juniper rusts (including cedar-apple rust, cedar quince rust, and cedar-hawthorn rust), which are all caused by fungal agents and their control is achieved mainly through chemical control (Sutton, 1996; Igarashi *et al.*, 2016). However, their inadequate or excessive use may contaminate the environment and harm human health. Furthermore, prolonged use of these products may result in selection pressure of the pathogenic agent, inducing the appearance of resistant isolates (Bonaterra *et al.*, 2012).

Plants possess natural defenses against a diverse range of pathogens. The *Malus* genus has coevolved with its pathogens, and many accessions harbor resistance genes that are exploited for introgression from wild relatives in apple breeding programs (Kumar *et al.*, 2010; Laurens *et al.*, 2011). The breeding of apples resistant to scab is a genetically-based strategy. Scab is the most important disease in terms of economic cost in apple orchards worldwide. *V. inaequalis* develops in the fallen leaves of apple trees. The infection starts in the spring and early summer. The pathogenic phase starts with the germination of ascospores (sexual spores), which serve as the primary source of inoculum. Conidia (asexual spores) serve as a source for secondary infection. Several cycles of conidial production and secondary infections occur within a single apple-growing season (MacHardy *et al.*, 2001). Scab causes deformation in the shape and size of the fruits, leaves, and premature fall of the fruit. The breeding programs follow, in general, a common strategy, which establishes a series of backcrossings between resistant hybrids and susceptible commercial cultivars. Usually, the source of resistance genes comes from sexually compatible wild apple relatives. This process is very time-consuming and demands cultivation of several generations to recover near-isogenic lines of high commercial value expressing the desired trait. In the apple, self-incompatibility turns breeding into an even more difficult endeavor as the elite varieties might not be recovered by crossing. As a result, strategies able to directly transfer traits to elite cultivated varieties would drastically accelerate the process.

## The genetics of scab resistance

The *V. inaequalis-Malus* interaction has been known for a long time (Boone and Keitt, 1957; Williams and Shay, 1957). Since the early days of apple resistance breeding, several sources of resistance genes have been described in the apple germplasms (Bus *et al.*, 2011).

The interaction between *V. inaequalis* and *Malus* species occurs in accordance to the gene-for-gene concept (Flor, 1971). Many resistance (R) genes have been identified in *Malus* mapping populations or from wild relatives among others (Jha *et al.*, 2009; Bus *et al.*, 2011). In this regard, the *Vf* locus of *Malus floribunda* was the first one to successfully undergo the process of introgression in *Malus* *domestica*. The *Vf* locus has been widely adopted in improvement programs directed towards the resistance against apple tree scab (Crosby *et al.*, 1992; Vinatzer *et al.*, 2001; Xu and Korban, 2002).

A cluster of four receptor-like sequences has been identified at the *Vf* locus, and these sequences are homologs to the *Cf* resistance genes from tomato. Three of the *Vf* genes, designated *Vfa1*, *Vfa2*, and *Vfa4*, have intact ORFs, whereas *Vfa3* is truncated and considered a pseudogene (Xu and Korban, 2002). The overexpression of the *Hcrvf2* (*Vfa2*) gene, under the control of the constitutive promoter CaMV35S, was sufficient to confer scab resistance to the susceptible apple cultivar Gala (Belfanti *et al.*, 2004). However, this resistance is specific to *V. inaequalis* races 1 to 5, but not towards race 6 (Jha *et al.*, 2009). It was also shown that when the *Vfa2* gene was expressed under the control of its own promoter, it conferred partial resistance to *V. inaequalis* in Galaxy and McIntosh cultivars (Xu and Korban, 2002). In addition, the *Vfa1* gene is capable of conferring partial resistance in susceptible cultivars races 1 to 5. This shows that these two genes are involved in resistance to *V. inaequalis*, while the *Vfa4* gene is not involved in resistance (Malnoy *et al.*, 2008).

Breeding programs have extensively studied resistance genes against apple scab worldwide. A new naming system was proposed based on the international standard *Arabidopsis* genes. The names of major *R* genes start with *R* and contain an abbreviation of the pathogen *V. inaequalis*, resulting in the locus general prefix *Rvi*. To date, 17 genes have been described: *Vg* (*Rvi1*) was identified in the cultivar Golden Delicious; *Vh2* (*Rvi2*) in TSR34T15; *Vh3* (*Rvi3*) in Geneva; *Vh4*, *Vx* and *Vr1* (*Rvi4*) in TSR33T239; *Vm* (*Rvi5*) in 9-AR2T196; *Vf* (*Rvi6*) and *Vfh* (*Rvi7*) in *Malus floribunda*; *Vh8* (*Rvi8*) in *M. sieversii*; *Vdg* (*Rvi9*) in K2; *Va* (*Rvi10*) in A723-6^b^; *Vbj* (*Rvi11*) in *M. baccata jackii*; *Vb* (*Rvi12*) in Hansen's baccata; *Vd* (*Rvi13*) in Durello di Forlì; *Vdr1* (*Rvi14*) in Dulmener Rosenapfel^b^; *Vr2* (*Rvi15*) in GMAL 2473; *Vmis* (*Rvi16*) in MIS op 93.051 G07-098^b^, and *Va1* (*Rvi17*) in Antonovka APF22^b^ [7] (Bus *et al.*, 2011).

## Transformation and genetic modification of apple

Traditional apple breeding is a slow process, because of the long juvenile phase and high degree of genetic heterozygosity present in this perennial fruit tree. For the breeding of almost all scab-resistant cultivars, the focus has been mainly on the *Vf2* gene (Gessler and Pertot, 2012). Once scab resistance still primarily relies on *Vf*, genes from other sources will be needed to overcome resistance breakdown over time. Alongside the classical breeding programs, in the past decades, several apple genetic modification strategies have been developed for obtaining scab-resistant varieties. Recent advances in genomics have allowed apple genetic transformation to become a promising technology for the acceleration of development of elite cultivars of apple (Gessler and Patocchi, 2007).

The resistance gene *HcrVf2/Rvi6* was transferred by *Agrobacterium tumefaciens* to susceptible apple cultivars (Barbieri *et al.*, 2003; Belfanti *et al.*, 2004; Silfverberg-Dilworth *et al.*, 2005; Malnoy *et al.*, 2008), and further attempts even led to the attainment of cisgenic resistant plants carrying *HcrVf2*/*Rvi6* (Joshi *et al.*, 2011; Vanblaere *et al.*, 2011, 2014) and not carrying any foreign DNA nor selection markers (Krens *et al.*, 2015). Several other scab resistance genes have been mapped; however, only *Vr2/Rvi15* (Bus *et al.*, 2011) has been functionally characterized, and this has conferred scab resistance to the ‘Gala’ cultivar (Schouten *et al.*, 2014).

Although these approaches have successfully delivered the introgression of the *Vf2* gene, the sole use of *Vf2* in scab-resistance breeding programs and in GM plants is not advisable. The appearance of new virulent fungal races is continuous in a perennial orchard, and combining diverse disease resistance mechanisms could help to improve disease management strategy. As stated by Gessler and Pertot (2012), the major bottleneck for new resistance breeding is the lack of cloned resistance genes in *Malus*. From the publication of the first draft genome of apple in 2010 (Velasco *et al.*, 2010), there is still much work remaining to associate resistance phenotypes to linkage groups, and finally to cloned resistance genes.

Many efforts have focused on the utilization of genes from other source species in order to confer more broad resistance traits not dependent on pathogen race recognition. Some attempts in this direction have been reported using heterologous genes, known for their antifungal activity, such as genes from the biocontrol fungus *Trichoderma atroviride* (formerly *T. harzianum*). This fungus produces many chitinolytic enzymes including endochitinase, which randomly cleaves chitin, a major component of the fungal cell wall. One *Trichoderma atroviride* endochitinase encoded by the *ech42* gene inhibits the germination of spores and hyphae elongation. One study obtained several transgenic lines of the scab-susceptible apple McIntosh cultivar with varying expression levels of the *ech42* gene (Bolar *et al.*, 2000). Some transgenic lines showed an increased resistance to *V. inaequalis* with constitutive expression of fungal endochitinase. However, these lines with high endochitinase activity displayed reduced plant vigor. Similar results were also observed when the *ech42* gene was introduced in Galaxy and Ariane apple cultivars alone or co-transformed with the *T. atroviride* exo chitinase *nag70* (Bolar *et al.*, 2001). This reduction in plant vigor seems to be related to the high lignin content and higher peroxidase and glucanase activities in the transgenic lines (Faize *et al.*, 2003).

The antifungal wheat protein puroinduline B (*pinB*), a member of plant lipid transfer proteins (LTPs), was expressed in Ariane (an apple cultivar carrying *Vf*), which is resistant to races 1-5 *V. inaequalis*, and Galaxy, which is susceptible to all races. In these experiments, both Galaxy and Ariane *pinB* overexpressors displayed enhanced resistance to scab (Faize *et al.*, 2004). This suggests that the combination of diverse resistance strategies can improve the field performance of these plants where they are exposed to a wide diversity of pathogens. Besides apple scab, genes conferring resistance to fire blight (Liu *et al.*, 2001; Borejsza-Wysocka *et al.*, 2007; Malnoy *et al.*, 2007; Broggini *et al.*, 2014), *Alternaria* blotch (Fan *et al.*, 2011), and powdery mildew (James *et al.*, 2004; Caffier and Parisi, 2007; Chen *et al.*, 2012) have been described, and these could be used in conjunction with scab resistant genes in order to obtain multiple resistance via gene pyramiding. Many genes could be readily tested in transient assays with virus-derived vectors instead of generating apple transgenic plants for testing each gene.

Despite that genetic and genomic studies have progressed to a very advanced level in apple, the application of the acquired knowledge for marker assisted breeding (MAB) remains limited to a few breeding programs, probably due to difficulties associated with its implementation (Bassil and Lewers, 2009). MAB requires both the development and application of a complex knowledge and access to well-equipped genotyping facilities. Therefore, MAB is being used mainly for pyramiding monogenetically inherited traits like fruit quality and disease resistance (apple scab, powdery mildew and fire blight) with the purpose of generating homozygous breeding elite lines that can be used in further crosses (Baumgartner *et al.*, 2015). During the course of DNA markers development in apple, scab resistance has preceded that of other traits and have evolved from dominant RAPD (Random Amplified Polymorphic DNA) markers (Koller *et al.*, 1994) to co-dominant microsatellites and, more recently, to Single-Nucleotide Polymorphism (SNP) markers (Patocchi *et al.*, 2009, Igarashi *et al.*, 2016, Jänsch *et al.*, 2015). In the last decade, genomic sequences and chromosome maps of various cultivars have become available, allowing the development of large SNP arrays (Bianco *et al.*, 2014, Bianco *et al.*, 2016, Baumgartner *et al.*, 2016), turning these markers into the best choice for high-throughput analysis, reducing the costs of MAB strategies. The SNP markers are expected to provide solutions to several problems of apple breeding such as to increase the precision of selection, the lengthy juvenility and the large field space required for growing populations. In order to advance the selection of new apple cultivars, another challenge is to shorten the long-term apple tree juvenility. This juvenile phase can last between 4 and 10 years. The first fast cycle breeding approach using the effective introgression of *BpMADS4,* a flowering gene from European silver birch (*Betula pendula* Roth.) resulted in an early flowering phenotype in ‘Pinova’ (Flachowsky *et al.*, 2011), ‘Gala’, ‘Mitchgla Gala’, and ‘Santana’ (Weigl *et al.*, 2015) cultivars. Besides, silencing of the floral repressor *MdTFL1 (TERMINALFLOWER1)* gene inhibits vegetative growth and accelerates flower development (Flachowsky *et al.*, 2012). The transformation and plant regeneration process is a lengthy and costly process, usually with a low efficiency rate, but many efforts have made it feasible in apple, with a large set of transgenic lines described (Aldwinckle and Malnoy, 2009). Commercial apple varieties are difficult to breed with new characteristics, as each time one introduces a new trait by crossing, a completely new genotype with new characteristics will be generated. Self-incompatibility in the apple does not allow simple backcrossings, and time-consuming pseudo-backcrossings lead to a delay in trait delivery. Consequently, biotechnological tools able to transfer resistance into already-cultivated cultivars would be an excellent opportunity to accelerate resistance breeding.

Moreover, if several genes have to be crossed in order to achieve durable resistance to multiple pathogens, then achieving such a multigenic combination into apple by conventional breeding becomes a nearly impossible task.

## Technological strategies using transient virus

An alternative method to achieve genetic transformation and transgenic plants for trait delivery relies on plant viruses. Apple latent spherical virus (ALSV) is an infectious plant virus capable of spreading through infected cells without inducing any diseases. Initially used for VIGS (Virus-Induced Gene Silencing) experiments in herbaceous plants, the ALSV vector is capable of sustaining uniform silencing phenotypes and persisting for several months (Igarashi *et al.*, 2009). Apple seedlings were inoculated with rbcS-ALSV, and the phenotype of systemic silencing persisted for more than three months in most of the infected plants (Sasaki *et al.*, 2011). Thus, the VIGS system using ALSV vectors can induce both VIGS and gene overexpression in apple seedlings as efficiently and persistently as in herbaceous plants (Yamagishi *et al.*, 2014). Furthermore, ALSV has been modified to express genes that confer desirable traits such as early flowering (Sasaki *et al.*, 2011; Yamagishi *et al.*, 2011). A construct of a modified ALSV vector (*ALSV-AtFT/MdTLF1-1*) designed to express the *Arabidopsis thaliana* florigen protein FLOWERING LOCUS T (*AtFT*) and to suppress the expression of TERMINAL FLOWER 1 (*MdTFL1-1*) was delivered by biolistic inoculation to germinated apple seeds where it was able to reduce the flowering period for approximately one year. In addition, it was verified that the ALSV vector was not transmitted to the majority of the successive progenies (Yamagishi *et al.*, 2014). This technique might have a major impact on the acceleration of breeding programs, as it does not involve the genetic modification of the host plant and allows both overexpression and silencing of trait genes. One of the major drawbacks of this system is the use of a full-length infectious virus that needs to be maintained and propagated in host plants. The insert size limitation is usually related to the carrying capacity of the viral genomes and, in addition, the method of administration relies on biolistics of the mature viral RNA, which can be methodologically laborious.

## New perspectives: plant episomal plasmids

During the past decade, many advances have occurred in the plant biotechnology field. For perennial plants, one of the most interesting and overlooked processes has been the expression system based on episomal plant plasmids. The system, called IL-60, is derived from the Tomato Yellow Leaf Curls Virus (TYLCV), a monopartite ssDNA geminivirus with a broad host range (Peretz *et al.*, 2007). IL-60 is based on a modified virus that can be mechanically administered to plants and has all of the symptom-causing ORFs removed or mutated to render a dsDNA plasmid able to be replicated in plant cells as an episomal plasmid. The viral regions essential for gene expression and cell-to-cell movement are maintained, and specific trait genes can be directly introduced into plants. The IL-60 plasmids will replicate, spread to other tissues, and eventually express the trait gene throughout the plant body in a stable manner for the full life cycle of the treated plant, and many species are compatible, including woody fruit trees such as grapevine, citrus, and olive. As observed for the ASLV vectors (Yamagishi *et al.*, 2014), the TYLCV-derived plasmids are not heritable, and the progeny will be devoid of the plasmid DNA. Further development of the system allowed the expression of a full bacterial operon in tomato plants (Mozes-Koch, *et al.*, 2012). The construction was somehow processed from a polycistronic RNA, and the metabolite pyrrolnitrin synthesized from the four introduced bacterial genes was detected by HPLC in the treated plants. The tomato plants able to synthesize the antifungal pyrrolnitrin displayed disease resistance to *Rhizoctonia solani*. The system has been updated, and only minimal regions of the TYLCV genome, essential for the maintenance and movement of the plasmids, were kept, resulting in two plasmids named pIR and p1470 (Gover *et al.*, 2014). An Israeli company named Morflora (http://www.moreflora.com/) retains a patent for the use of the IL-60 plasmids by the name of TraitUP^TM^.

This technique was already successfully tested in apple with transient assays for the characterization of *Malus* COP1 orthologs (Li *et al.*, 2012), which opens an alternative for the functional analysis of apple genes in a homologous system. From our side, we have performed successful preliminary tests using apple plants and the IL-60 plasmids for expression of reporter genes such as GFP. Apple plants from the Gala cultivar treated with pIR-GFP plus p1470 displayed a broad, stable, and strong GFP expression that spread through all tissues progressively and remained stable even six months after treatment with the plasmid DNA (data not shown). The compatibility of such plant plasmids with apple allows us to use it as a genetic platform for transferring scab resistance genes (such as *Vf2*), as well as other genes of biotechnological interest, to elite apple cultivars. The genes seem to be stably expressed throughout the plant's development. This method eliminates the need for crosses and plant transformation turning into an immediate genetic trait transfer tool.

One exciting new possibility for genome editing and trait introgression appeared after the discovery of CRISPRs/Cas9 (Jinek *et al.*, 2012; Cong *et al.*, 2013) nucleases, which need to be transiently expressed only to create site-specific double strand breaks (DSBs). Plant episomal plasmids can be engineered to deliver these tailored nucleases to plants, as described for *Agrobacterium* binary vectors (Baltes *et al.*, 2014; Kumar and Jain, 2014; Ali *et al.*, 2015), and gene-specific modifications can be obtained without creating a transgenic plant. Recently, successful targeted mutagenesis has been obtained in plants with DNA-free preassembled CRISPR-Cas9 (Woo *et al.*, 2015), which would avoid the generation of transgenic cells and accelerate product development. A significantly increased availability of genomic information for fruit species expands this possibility among many cultures, and a feasible expression system is essential for the application of these techniques (Yin *et al.*, 2015). Many relevant traits in apples are dependent on loss-of-function mutations, such as ripening (Dandekar *et al.*, 2004), self-incompatibility (Broothaerts *et al.*, 2004), allergenicity (Gilissen *et al.*, 2005), and fire blight resistance (Borejsza-Wysocka *et al.*, 2004), among many others (Bulley *et al.*, 2007). Recently, engineered point-mutations in the COI1 JA co-receptor rendered coronatine-insensitive *Arabidopsis* plants showing enhanced resistance to pathogenic bacteria (Zhang *et al.*, 2015). A proof-of-concept study utilizing CRISPR/Cas9 introduced recessive mutations in *Arabidopsis* *eIF(iso)4E* locus for conferring resistance to *potyvirus* (Pyott *et al.*, 2016). Similar mutations could be delivered by transient expression of tailored site-specific nucleases into the apple breeding germplasm to develop more scab-resistant cultivars.

The new developments achieved in the recent past years have opened exciting new possibilities for fast trait delivery in woody fruit species (Nagamangala Kanchiswamy *et al.*, 2015). Besides genetic transformation, which has been very successful in apple despite the hurdles of plant regeneration, easier, faster, and readily approachable new options are available for breeders worldwide. The long juvenile cycles, the large population screens, the high-throughput phenotyping/genotyping, and successive crossings are still essential to any breeding program, but we can accelerate and facilitate the process with the technologies that are just waiting to be exploited. These episomal expression systems, based on modified viruses, may enable the expression of stable traits that can be introduced by treating scions prior to grafting using elite genotypes, bypassing the backcrossing events to recover the original genetic background. Concerns regarding biosafety and transgene escapes are still to be assessed, but due to the lack of heritability of the episomal DNA, this control might be easier than pollen-mobile genetic contamination derived from genetically modified plants.
